# Prolonged Use of an Impella Assist Device in a Sepsis-Induced Cardiomyopathy: A Case Report

**DOI:** 10.7759/cureus.18889

**Published:** 2021-10-19

**Authors:** Ala Mustafa, Jacob Obholz, Nathaniel Hitt, Richard Rattin

**Affiliations:** 1 Internal Medicine, MercyOne North Iowa Medical Center, Mason City, USA; 2 Interventional Cardiology, MercyOne North Iowa Medical Center, Mason City, USA

**Keywords:** impella cp, cardiogenic shock, severe sepsis, assist device, cardiomyopathy

## Abstract

The inflammatory response triggered by sepsis can frequently cause reversible myocardial depression termed sepsis-induced cardiomyopathy. The resulting pathologic changes are often self-limiting and cardiac function returns to baseline following resolution of the underlying exacerbating factors. The following case examines a patient with septic shock and sepsis-induced cardiomyopathy that, despite maximal medical therapy, required mechanical support with an Impella assist device for seven days. To the best of our knowledge and research, this represents the longest documented use of an Impella heart pump in septic shock and associated sepsis-induced cardiomyopathy. Utilization of mechanical support in the setting of septic shock has seen growing interest in recent years, but more structured studies need to be conducted for better understanding of their overall effect on morbidity and mortality.

## Introduction

Cardiogenic shock commonly presents as profound hypotension and reduced cardiac output leading to pulmonary edema, elevated central venous pressure (CVP) (>12 mmHg) and low SvO2 (<70%) [1]. Large dilated ventricles and reduced left ventricle function can be seen on echocardiography. Hypoperfusion becomes evident when systolic blood pressure is less than 90 mmHg or the mean arterial pressure is less than 70 mmHg [1]. Decreased perfusion and vasoconstriction clinically present as cold, clammy extremities, decreased urinary output, and confusion. The etiology of cardiogenic shock often involves mechanical dysfunction secondary to acute myocardial infarction or acute mitral regurgitation. However, other etiologies include underlying sepsis causing cardiomyopathy [2]. Some of these patients may require mechanical circulatory support [1]. One such support system is the percutaneous left ventricular assist device (Impella) which aids in the circulation of blood from the left ventricle into the ascending aorta. Common indications for the use of an Impella include cardiogenic shock as a result of acute myocardial infarction, open heart surgery, or in setting of acute left heart failure in preexisting cardiomyopathy [3]. Several cases report the use of the Impella device in myocardial depression and cardiogenic shock secondary to sepsis, however all reports we could identify were for no more than 4.5 days in duration [4]. We present the case of a patient who developed cardiogenic shock due to sepsis and was treated with seven days of mechanical circulatory support with an Impella device CP. The patient subsequently improved and was without complication. To our best knowledge and research, this represents the longest reported time of an Impella device used for a patient with sepsis-induced cardiomyopathy.

## Case presentation

An 89-year-old male was admitted to the intensive care unit (ICU) due to hypotension with systolic blood pressure <70 mmHg and notable positive blood cultures and urinalysis. The patient had a past medical history of hypertension, hyperlipidemia, coronary artery disease, inferior myocardial infarction requiring intervention, diastolic heart failure with an ejection fraction of 50%, diabetes mellitus type 2, metastatic prostate cancer, periampullary carcinoma, and multiple myeloma. On presentation, the patient had cold extremities, confusion, and reported difficult urinating. Labs showed a high sensitivity troponin 25.6 pg/ml, lactate 2.16 mg/dl, brain natriuretic peptide (BNP) 239 pg/ml, and leukocytosis 10.79 x 10^3^/ul. The urinalysis showed 2+ leukocyte esterase with 26-50 white blood cells and the blood cultures were positive for gram negative rods. Antimicrobial therapy targeting septic shock with a urinary source was started. However, he began to complain of substernal and epigastric pain accompanied by respiratory failure requiring intubation and mechanical ventilation. Repeat labs showed a high sensitivity troponin 1,144 pg/ml, lactate 5.8 mg/dl and leukocytosis 17.50 x 10^3^/ul. Transthoracic echocardiography showed a reduced left ventricular ejection fraction of 25-30% as seen in Video 1. The patient was started on escalating doses of norepinephrine, vasopressin, hydrocortisone, and dobutamine. However, despite maximum medical therapy, hypotension persisted, and renal failure developed requiring the initiation of dialysis. Cardiology was consulted and the patient was diagnosed with a type 2 myocardial infarction and sepsis-induced cardiomyopathy. The patient was started on guideline non-ST-segment elevation myocardial infarction (STEMI) medications and taken to cardiac catherization lab where an Impella device CP and a pulmonary artery catheter were placed. A pulmonary arterial catheter was also placed which showed a cardiac output of 10.61 l/min, cardiac index 5.1 l/min/m^2^ and systemic vascular resistance of 3.83. By day 5 of hospitalization, the patient was no longer requiring vasopressor support. The Impella device was removed on the patient’s seventh day in the hospital and the patient was extubated five days later. Repeat transthoracic echocardiography showed improvement in patient's left ventricular systolic function to 35-40% as seen in Video 2. The patient was stepped down to the medical floor and was eventually discharged from the hospital.

**Video 1:** Transthoracic echocardiography showed a reduced left ventricular ejection fraction of 25-30%

**Video 2:** Repeat transthoracic echocardiography showed improvement in patient's left ventricular systolic function to 35-40%

## Discussion

Two distinct cardiomyopathies are seen in septic shock, sepsis-induced cardiomyopathy and stress cardiomyopathy. Sepsis-induced cardiomyopathy is typically a reversible myocardial depression which occurs in the setting of septic shock. Although not fully understood, it is postulated that the myocardium is injured by inflammatory cytokines, endotoxins, and nitric oxide leading to mitochondrial dysfunction, decreased myofibril response to calcium ions, and downregulation of B-adrenergic receptors. Sepsis-induced cardiomyopathy is defined by three distinct criteria: left ventricular dilation, depressed ejection fraction, and recovery in 7-10 days. Left ventricular dilation and depressed ejection fraction are easily detected by echocardiography making it the most important diagnostic test [5].

In comparison, stress cardiomyopathy is likely due to an increased amount of catecholamines caused by sympathetic hyperactivity secondary to increased stress such as sepsis rather than the postulated inflammatory response seen in sepsis-induced cardiomyopathy [6]. Another differentiating feature between the two conditions is the degree of ventricular dysfunction. Sepsis-induced cardiomyopathy is associated with global ventricular dysfunction while stress cardiomyopathy has regional dysfunction which results in an apical ballooning pattern on echocardiography. Both conditions tend to cause reversible damage to the myocardium, however the recovery period associated with stress cardiomyopathy is stereotypically longer taking several weeks [5,6]. Our case demonstrated global ventricular dysfunction and an initial recovery within one week consistent with sepsis-induced cardiomyopathy.

Although pathologically different, the optimal treatment of the two disease states is similar due to their reversible nature. Both require controlling the underlying infection and providing hemodynamic support during the recovery period [5]. According to the Surviving Sepsis Campaign 2016 guidelines, norepinephrine is the first-line vasopressor with vasopressin as an appropriate adjunctive medication. Current guidelines recommend the use of dobutamine for further ionotropic support (Grade 1C), but recent studies question its benefit [5, 7]. Although dobutamine increases the cardiac index, it has not been shown to reduce mortality even in cases with severe heart failure (Ejection fraction <35%) [8]. One 2013 study suggested that it may even be associated with an increase in 90-day mortality [9].

Mechanical support in the setting of septic shock has recently seen growing interest. In isolated case studies, intra-aortic balloon pumping and veno-arterial extracorporeal membrane oxygenation (ECMO) have shown promising preliminary results [5]. Left ventricular assist devices are more widely available when compared to ECMO and, therefore, more likely to be utilized as was the case in our patient. An alternative ventricular support system to an intra-aortic balloon pump and ECMO is an Impella device. This may be a potential adjunctive therapy in patients, like ours, with sepsis-induced cardiomyopathy. Studies have shown the Impella device to be non-inferior to the intra-aortic balloon pump in cardiogenic shock and to even provide more hemodynamic support with no difference in clinical outcomes [4, 10]. Studies have also shown that placement of an Impella device can improve left ventricular output when medications alone failed to improve hemodynamic instability [4]. The device is placed in the left ventricular outflow tract using a catheter with access at the femoral or subclavian artery. It assists in increasing cardiac output by pumping blood from the left ventricle to the aorta at a rate of 2.5 to 5 L/min regardless of any concurrent arrythmias that may be present [3]. Additionally, this lowers cardiac workload by unloading the left ventricle, giving the heart time to recover. The current indications for the Impella device are sudden onset cardiogenic shock following a myocardial infarction or heart surgery, facilitation of high-risk coronary angioplasty, acute decompensation of cardiomyopathies, and post-cardiotomy shock [3]. Given these indications it is imperative to say that this was an "off-label" use of this device, however given the severity of this patient's condition, the decision was made to deploy this device while allowing the patient to recover. The successful recovery of our patient after placement of the Impella device suggests it may also have a role in sepsis-induced cardiomyopathy.

## Conclusions

This report demonstrates the longest use of an Impella heart pump in the management of sepsis-induced cardiomyopathy without serious complications. It suggests mechanical support with an Impella CP device may be an effective adjunctive treatment for sepsis-induced cardiomyopathy. However, the effectiveness, complications, morbidity, and mortality associated with this treatment requires additional research. The use of mechanical intervention in these critical patients may have a promising future, however more research is required to better define their true utility.
